# Recent Advances of Constructing Metal/Semiconductor Catalysts Designing for Photocatalytic CO_2_ Hydrogenation

**DOI:** 10.3390/molecules28155693

**Published:** 2023-07-27

**Authors:** Zhimin Yuan, Xianglin Zhu, Zaiyong Jiang

**Affiliations:** 1 School of Chemistry & Chemical Engineering and Environmental Engineering, Weifang University, Weifang 261061, China; 2 Institute for Energy Research, School of Chemistry and Chemical Engineering, Jiangsu University, Zhenjiang 212013, China

**Keywords:** photocatalytic CO_2_ reduction, metal/semiconductor, surface plasmon resonance, photocatalysts

## Abstract

With the development of the world economy and the rapid advancement of global industrialization, the demand for energy continues to grow. The significant consumption of fossil fuels, such as oil, coal, and natural gas, has led to excessive carbon dioxide emissions, causing global ecological problems. CO_2_ hydrogenation technology can convert CO_2_ into high-value chemicals and is considered one of the potential ways to solve the problem of CO_2_ emissions. Metal/semiconductor catalysts have shown good activity in carbon dioxide hydrogenation reactions and have attracted widespread attention. Therefore, we summarize the recent research on metal/semiconductor catalysts for photocatalytic CO_2_ hydrogenation from the design of catalysts to the structure of active sites and mechanistic investigations, and the internal mechanism of the enhanced activity is elaborated to give guidance for the design of highly active catalysts. Finally, based on a good understanding of the above issues, this review looks forward to the development of future CO_2_ hydrogenation catalysts.

## 1. Introduction

The world’s consistent and large-scale dependence on fossil fuels during the past hundred years has had serious repercussions, including energy crises and the deterioration of the global climate, which was caused primarily by increasing atmospheric concentrations of CO_2_ [1,2,3,4,5,6,7,8,9]. Though it has historically been discharged directly into the atmosphere as waste, increasing global interest in transitioning away from the use of fossil fuels now forces us to change our previous conceptions by employing carbon dioxide as a recyclable feedstock for the production of renewable synthetic fuels [10,11,12,13,14,15,16,17]. In recent decades, several methods have developed for carbon dioxide reduction, for example, electrocatalytic [18,19,20,21], photocatalytic [22,23,24,25,26,27,28], photoelectrochemical (PEC) [29,30,31,32], thermocatalytic [33,34,35,36,37], and photothermal [38,39,40] reduction, etc. These mentioned methods can be driven by clean electric energy or solar energy; therefore, they are considered the most promising strategies. In the case of electrocatalytic reduction [41,42,43,44], the two key issues facing electrocatalytic CO_2_ reduction are obtaining an inexpensive and a renewable source of electrical energy and the development of highly active CO_2_ reduction electrocatalysts. As a result, photovoltaic cells are typically used as the electricity source (thereby allowing for more direct energy conversion from sunlight), and carefully designed electrocatalysts form the cathodes on which CO_2_ reduction takes place. Though much research has been conducted to address these issues, high overpotentials, poor selectivity, low electric current densities, catalyst instability, and relatively high costs of renewable electric energy continue to limit the large-scale application of electrocatalytic CO_2_ reduction [45]. Photocatalytic CO_2_ reduction offers another appealing strategy for reducing CO_2_ into fuels by also directly absorbing solar energy as a source of energy while employing water as an electron donor [46,47,48,49]. Due to the limitations of low separation efficiency for photogenerated carriers and slow reaction kinetics in both CO_2_ reduction and H_2_O oxidation, the highest product formation rates reach only micromoles per gram of catalyst per hour (μmol g_cat_^−1^ h^−1^) [50]. To overcome those limitations, many studies have been trying to embed the donor–acceptor scheme into the primary photosensitizing module [51,52,53,54,55,56]. By comparison, thermocatalytic CO_2_ reduction has proven an efficient approach for the hydrogenation of CO_2_ to form methanol (CH_3_OH), methane (CH_4_), carbon monoxide (CO), and more at high rates using heterogeneous catalysts [57,58,59,60,61,62]. However, these processes require higher reaction temperatures (e.g., 200 to 250 °C) and high pressures (5 to 10 MPa) than the previously mentioned methods. Such high temperatures can be disadvantageous, though, when synthesizing products, such as methanol, whose high reaction exothermocity results in diminishing maximum theoretical yields at higher temperatures [63,64]. A related technology, solar thermal catalysis, has also emerged as a promising approach for the synthesis of renewable fuels from CO_2_, combining the efficacy of thermocatalysis with a clean source of energy (focused sunlight).

Compared to the electrocatalytic, photocatalytic, and pure thermocatalytic approaches introduced previously, solar thermal reduction methods utilize energy from a large range of the solar spectrum (making use of materials exhibiting intense, broadband light absorption), while also offering high product selectivity relative to photochemical and electrochemical paths [65]. In light of these advantages, solar thermal catalysis has demonstrated its potential for use in industrially relevant chemical reactions, such as reducing nitrogen to ammonia (Haber process) [66], methane reforming [67], and preparing synthesis gas [68,69]. Though there have been many recent discoveries and developments regarding solar thermal catalytic reactions, these specific examples are highlighted for being significantly endothermic; as such, they benefit greatly from the high temperatures and thermal input generated by solar thermal systems.

## 2. Application of Metal/Semiconductor Catalysts for Photocatalytic CO_2_ Hydrogenation

The solar energy utilization efficiency in photocatalytic processes is determined by both the incident quantum absorption capability of the catalyst and its internal quantum yield. As a result, expanding the range of wavelengths absorbed by a catalyst has been proven to be one of the most effective ways of enhancing catalytic activity, especially for the semiconductor-based materials. Though great attention has been paid to broadening optical absorption ranges, such as introducing dopants, defects, sensitizers, or plasmonic metal, these strategies often result in unintended side effects (weakened redox capacity, decreased internal quantum yield, etc.). Thus, it is necessary to further explore novel catalytic systems and new mechanisms that can maximize both the light absorption efficiency and internal quantum yield [70]. Loading noble metal nanoparticles onto photocatalysts can enhance energy harvesting from visible-light photons, thanks to high-energy (“hot”) electrons generated by localized surface plasmon resonance (LSPR) [71,72]. The design of metal/semiconductor catalysts has been one potential strategy for the preparation of efficient photocatalytic CO_2_ hydrogenation catalysts.

### 2.1. Pd-Based Catalysts

**Figure 1:**
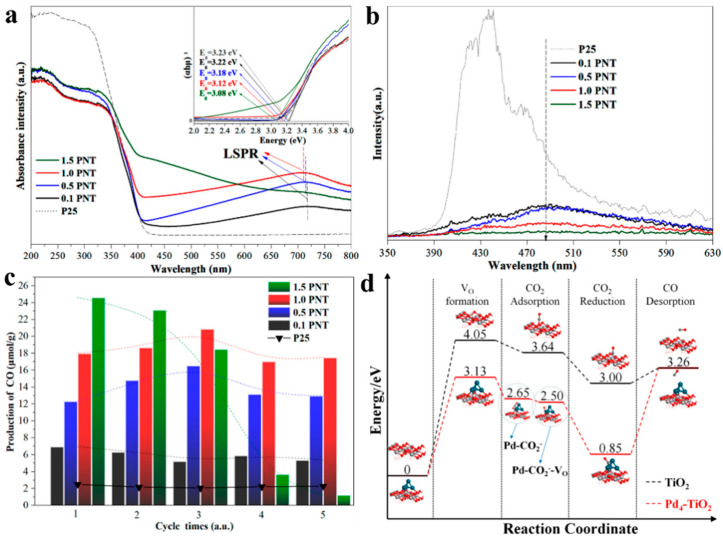
(**a**) Diffuse reflectance UV-vis spectra of original P25 and PNT samples (inset: determination of the optical band gap). (**b**) Photoluminescence spectra of P25 and the PNT samples. (**c**) CO yields obtained from five successive PTC runs. All samples were illuminated for 50 min under a He atmosphere and then heated at 773 K for 50 min under a CO_2_ atmosphere (The dashed line represents the changing trend of CO yield for each catalyst.). (**d**) DFT-calculated reaction pathways proceeding on P25 and PNT catalysts [73].

To lower the temperature needed in a thermocatalytic CO_2_ reduction and increase solar energy utilization, Ni et al., fabricated a palladium-nanoparticle-loaded TiO_2_ (PNT) catalyst employed using a photothermochemical cycle (PTC) method [73]. In this system, the uniformly dispersed Pd nanoparticles achieved LSPR absorption in the visible region. As a result, the PNT exhibited enhanced light utilization thanks to its red-shifted absorption range in the visible spectrum (Figure 1a). Absorbed visible IR light could then be converted to thermal energy and incorporated into a chemical reaction, allowing the 1.0 PNT catalyst to realize a stable CO production rate of 11.05 μmol g_cat_^−1^ h^−1^ (more than eight times the CO production rate of P25 using the PTC) (Figure 1c). The structure of the PNT catalyst also allowed the electron–hole pairs generated from the photoexcitation of TiO_2_ to be more efficiently separated (Figure 1b), thereby increasing the number of available charge carriers. Thus, the reduction rate of CO_2_ production can be accelerated by an advantage of photothermal synergy. Complementary density functional theory (DFT) calculations indicated that Pd can play a further role in promoting CO_2_ adsorption by forming Pd-CO_2_^−^ and Pd-CO_2_^−^-V_O_ at defect sites during the thermal catalytic progress (Figure 1d).

**Figure 2:**
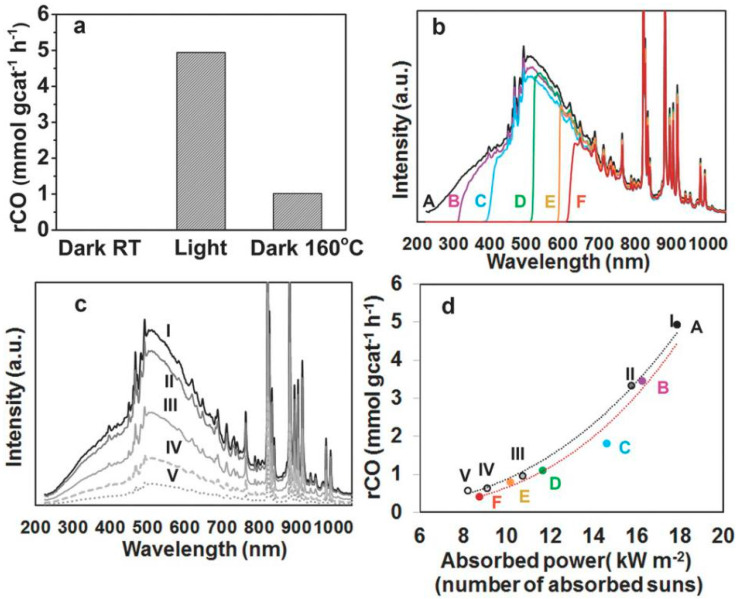
(**a**) Photothermal CO production rates over Pd@Nb_2_O_5_. (**b**) Spectral irradiance incident on the Pd@Nb_2_O_5_ catalyst with different cut-off filters (A through F). (**c**) Spectral irradiance incident on the Pd@Nb_2_O_5_ catalyst for batch reactions I through V. (**d**) RWGS reaction rates plotted as a function of absorbed power for the series of batch reactions A through F (red line) and *I* through *V* (black line) [74].

Another promising series of photothermal catalysts was developed by Ozin et al., using Pd to provide deeper insight into the synergistic mechanisms responsible for the highly selective reduction in CO_2_. In the first such work, nanostructured Pd@Nb_2_O_5_ nanorod catalysts were prepared via an impregnation method and tested in the gas-phase hydrogenation of CO_2_ to CO [74]. The use of ethanol as a reducing agent was instrumental in forming uniformly small Pd nanoparticles on the Nb_2_O_5_ nanorods, as it allowed the reduction reaction rate of the palladium precursor to be controlled, thereby suppressing particle formation. Experiments proved that the Pd nanoparticles were well dispersed on the surface of the Nb_2_O_5_ nanorods and had diameters ranging from 2 to 10 nm. Due to their size, these Pd nanocrystals were able to absorb shorter wavelengths of light (visible and near-infrared (NIR) light) by exciting inter-band and intra-band electronic excitations, helping to initiate CO_2_ reduction. This increased amount of thermal energy was beneficial, as it was also proven that the reaction rate was significantly enhanced by the generation of Nb^4+^ and oxygen vacancies in situ (on reduction by H_2_). The Nb_2_O_5_ nanorods also featured a large surface area (as measured via BET gas adsorption), which provided a large number of CO_2_ adsorption sites for the reaction. Wavelength- and intensity-dependent catalytic activity measurements performed on the nanostructured Pd@Nb_2_O_5_ catalyst (Figure 2) revealed that the reverse water–gas shift (RWGS) reaction was activated photothermally, depending primarily on the conversion on light to thermal energy rather than the formation of electron–hole pairs. By virtue of this combination of features, the RWGS reaction can be catalyzed by the Pd@Nb_2_O_5_ catalyst under visible and NIR light even without external heating, achieving an impressive reaction rate of 1.8 mmol g_cat_^−1^ h^−1^.

**Figure 3:**
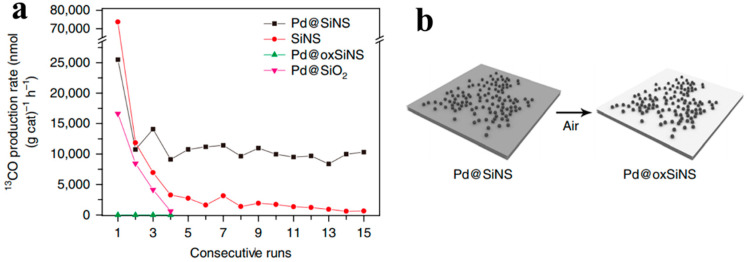
(**a**) ^13^CO production rate of four samples: Pd@SiNS, pristine SiNS, Pd@oxSiNS, and Pd@SiO_2_. (**b**) Preparation of the cAontrol sample Pd@oxSiNS via oxidation in air [40].

Silicon-based support materials have also been used in conjunction with ultrasmall (19-atom) Pd nanoparticles, which were deposited onto two-dimensional silicon–hydride nanosheets (Pd@SiNS) and used to catalyze the RWGS reaction. Due to the innovative synthesis method, Pd precursors were reduced in situ on the surface of the Si nanosheets, resulting in highly dispersed and well-immobilized Pd nanoparticles while also retaining the large surface area (197 m^2^ g^−1^) of the two-dimensional Si nanosheets. Several characterization techniques were also employed, including Fourier transform infrared spectroscopy, in situ isotopic labeling, and theoretical calculations to understanding the internal catalytic mechanism. [40] The Pd@SiNS sample showed a ^13^CO production rate of 10 μmol g_cat_^−1^ h^−1^, which was much better than that of the pristine SiNS sample (Figure 3a), and retained its good stability under repeated cycling (relative to pristine SiNSs). A crucial supporting experiment showed that Pd nanoparticles on fully oxidized Si nanosheets (Pd@oxSiNS) had no activity in CO_2_ reduction (Figure 3b), demonstrating the limited activity of Pd nanoparticles alone and proving the synergetic effect between Pd and the Si nanosheets. By studying these experimental results, a clear and fundamental understanding was reached, in that the H-transfer process from Pd to the oxidized SiNS surface represented two mechanisms. First, one H atom adsorbed onto a Pd nanoparticle interacted with a surface Si-O-Si site of the SiNSs. Resulting in the formation of a surface Si–OH site. Second, another active H from the Pd nanoparticle forms a bond with the generated Si-OH, leading to the desorption of a molecule of water and the formation of a highly reactive surface Si radical site capable of reacting with CO_2_ and enabling a catalytic reaction cycle. The strain induced in the SiNS surface by the Si-O-Si bonds is believed to be responsible for the enhanced reactivity of the oxidized SiNS surface toward catalytic hydrogenation of CO_2_ under mild conditions. This work also highlights the potential role of Si surface chemistry in designing and developing new heterogeneous CO_2_ catalysts in the future.

Similar synergistic mechanisms between metal co-catalysts and metal oxide supports were also presented by Li et al., in which a catalyst consisting of palladium nanoparticles deposited into tungsten–bronze nanowires (Pd@H_y_WO_3−x_) was used to efficiently catalyze the reduction in CO_2_ to CO at a rate of 3.0 mmol g_cat_^−1^ hr^−1^ with a selectivity exceeding 99% [75]. WO_3_ nanowires loaded with Pd nanocrystals provided the synthetic pathway to obtaining Pd@HyWO_3−x_. Through various characterization techniques, it was demonstrated that H_y_WO_3−x_ was formed via spillover of activated H from the Pd nanoparticles. The existence of Brønsted acid hydroxyls (-OH), W(V) sites, and oxygen vacancies (V_O_) in HyWO_3−x_ all contributed to the CO_2_ capture and reduction reactions.

### 2.2. Ru-Based Catalysts

**Figure 4:**
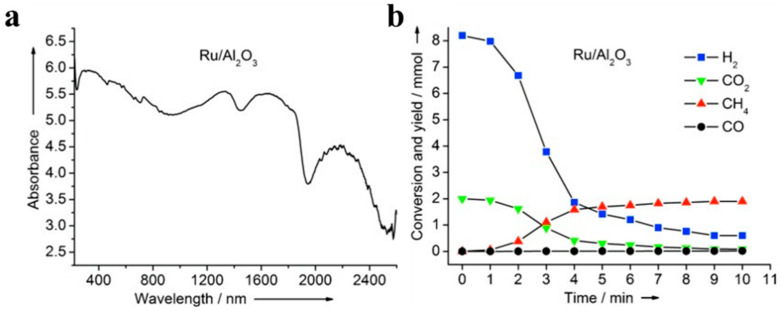
(**a**) Representative UV-Vis–NIR spectrum and (**b**) product yields of a metal/metal oxide photothermal nanocatalyst (Ru/Al_2_O_3_) [36].

Ye et al., prepared a series of Group VIII metals (Ru, Rh, Ni, Co, Pd, Pt, Ir, and Fe) loaded onto Al_2_O_3_ or TiO_2_ to form classical metal/metal oxide nanocatalysts and studied their photothermal effects on the catalytic reduction in CO_2_ into CH_4_ and CO (using H_2_) [39]. The loading of Group VIII metals onto Al_2_O_3_ or TiO_2_ resulted in highly active materials that had a dark grey or black color (Figure 4a) due largely to their nanoscale structure. A high product selectivity (over 95%) for either CH_4_ or CO could be achieved by adjusting the metal and support materials used in a catalyst (Figure 4b). The outstanding CO_2_ conversion ability of the prepared catalyst was attributed to two main features: highly efficient utilization via photothermal processes and the unique ability of the Group VIII metals to activate CO_2_ hydrogenation. Compared to pure photocatalytic pathways, photothermal CO_2_ conversion is not restricted to utilizing ultraviolet irradiation, as it can readily absorb energy from visible and infrared wavelengths (this range contains 96% of the solar energy) can utilized by photothermal CO_2_ conversion. In related research from the same group, Ru-loaded ultrathin, layered double hydroxides (Ru@FL-LDHs) were also prepared, achieving efficient photothermal CO_2_ methanation in a flow reactor system [76]. These catalysts were composed of well-dispersed Ru nanoparticles embedded in ultrathin, exfoliated LDH sheets. Under light irradiation, a maximum CH_4_ production rate of 277 mmol g_cat_^−1^ h^−1^ or 230.8 mol h^−1^ m^2^ (irradiation area) was attained, which was significantly higher than most reported LDH-based catalysts [77,78,79]. These experiments proved that ultrathin LDH sheets can provide an abundance of active sites for the adsorption and activation of CO_2_ molecules, allowing for the use of a high flow rate of feedstocks without decreasing the percent conversion of CO_2_ to products. Meanwhile, the highly dispersed Ru nanoparticles contributed by providing higher local surface temperatures under light irradiation (via photothermal heating) and activating H_2_ molecules, thereby promoting the hydrogenation of CO_2_. In this example, the noble metal particles and supporting LDH matrix activated the CO_2_ and H_2_ reagents separately, highlighting the potential benefits of designing catalysts by targeting the activation of individual reactant molecules.

In a related work by Dai et al., a Ru/TiO_(2−x)_N_x_ catalyst was prepared by loading Ru onto nitrogen-doped TiO_2_ support via an impregnation–reduction method [80]. The catalytic CO_2_ methanation performance of this catalyst was evaluated under both visible light irradiation (435 nm < λ < 465 nm) and under dark conditions in a fixed-bed flow reactor. With the illumination of visible light, the turnover frequency (TOF) of the catalyst increased to 15.0 h^−1^ (compared to the 7.0 h^−1^ under dark condition), with the CH_4_ selectivity remaining stable at about 80% throughout the duration of the reaction. A combination of chemisorption, surface analysis, and photocurrent measurements revealed that visible light contributed to the catalytic reaction in two ways. First, a greater number of oxygen vacancies were formed due to the stronger visible light response of TiO_2−x_N_x_, allowing CO_2_ to be more efficiently adsorbed, activated, and converted into the CO. Second, Ru nanoparticles can accept photogenerated electrons from TiO_(2−x)_N_x_, increasing the surface electron density of Ru nanoparticles and promoting the adsorption and activation of CO_2_.

**Figure 5:**
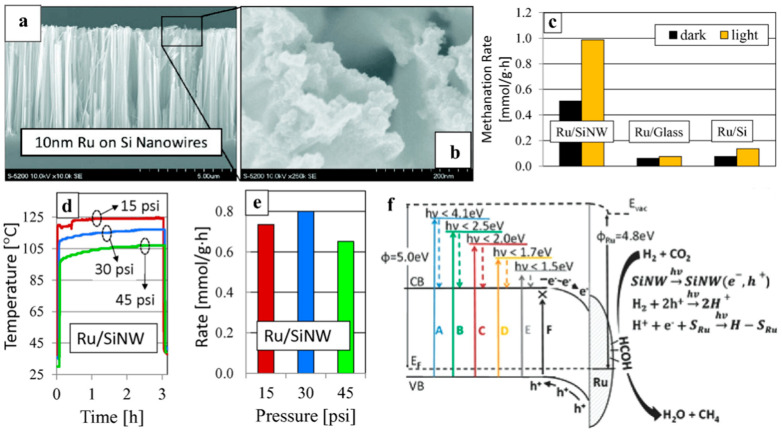
(**a**,**b**) Scanning electron microscopy (SEM) images of 10 nm Ru nanoparticle/silicon nanowires. (**c**) Methane production rates over Ru-based catalysts on SiNW, glass, and polished Si supports (**d**) Temperature profiles recorded for batch reactions performed at a different reaction condition and (**e**) methanation rates of a different sample. (**f**) The proposed energy band diagram at the SiNW-Ru interface and schematic diagram [81].

In another study from the same group, Ru/silicon nanowire catalysts (Ru/SiNW) (Figure 5a,b) were prepared by forming 10 nm Ru on silicon nanowires (SiNWs) via sputtering. In this case, the coexistence of Ru and SiNWs resulted in the possibility of activating the Sabatier reaction (CO_2_ + H_2_ → CH_4_ + H_2_O) both thermochemically and photochemically [81]. Detailed investigations were performed in the presence and absence of light irradiation (Figure 5c), with varying atmospheric pressure, (Figure 5d,e) and using varying wavelengths of light (Figure 5f) to better understand the internal mechanisms responsible for the observed catalytic activity. The results of these investigations revealed that the observed CO_2_ reduction mechanism could be predominantly photochemical or thermochemical, depending on the wavelengths of irradiating light. When the photon energy was less than the bandgap energy of silicon, the Ru/SiNW catalyst relied upon photothermal heating to activate the Sabatier reaction thermochemically. When the incident photon energy is sufficient to overcome the bandgap of Si, however, electron–hole pairs generated in the Ru/SiNW catalyst assisted the formation of R-H bonds. Eventually, a proportion of these excited charge carriers eventually thermalize and recombine, generating heat and supporting the thermochemical promotion of the Sabatier reaction. In this way, the prepared Ru/SiNW catalysts achieved a CH_4_ production rate of 0.74 mmol g_Ru_^−1^ h^−1^ with a simulated solar light intensity of 14.5 suns, a 4:1 H_2_/CO_2_ ratio, and an atmospheric pressure of 15 psia. The concepts and the experimental approaches introduced in this work are significantly helpful for understanding the synergistic contributions of photochemical and thermochemical mechanisms to the overall process of photothermal CO_2_ reduction.

**Figure 6:**
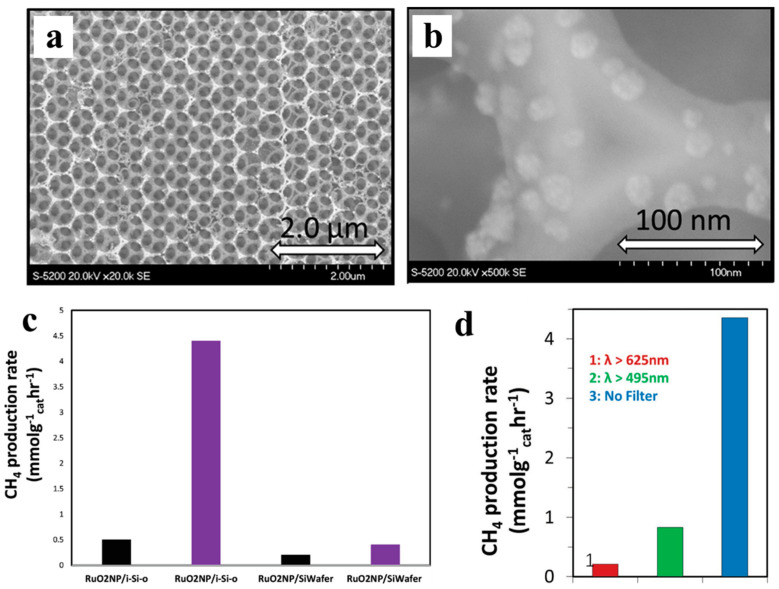
(**a**,**b**) SEM secondary electron images of ncRuO_2_/i-Si-O sample. (**c**) ^13^CH_4_ production rate from ncRuO_2_/i-Si-O and ncRuO_2_/Si wafer hybrid samples. (**d**) Photomethanation rates over the ncRuO_2_/i-Si-o catalyst tested under different illumination conditions [82].

In yet another study, the photothermal reduction in gaseous CO_2_ over Ru/silicon photonic crystal photocatalyst (Figure 6a,b) was studied at ambient temperature [82]. Ru/i-Si-o catalysts showed increasing activity as light intensity increased, achieving peak rates of up to 4.4 mmol g_cat_^−1^ h^−1^ (Figure 6c). The high photomethanation rates over the Ru/i-Si-o catalyst are attributed to the excellent light-harvesting properties of the silicon photonic crystal, as proven using control experiments (Figure 6d). Complementary simulation results further indicated that the charged Ru surfaces are beneficial for CO_2_ destabilization–adsorption progress; additionally, the charged Ru surfaces are crucial for the adsorbing and dissociating of H_2_, which can react with CO_2_ to accelerate the Sabatier reaction.

### 2.3. Other Metal-Based Catalysts

**Figure 7:**
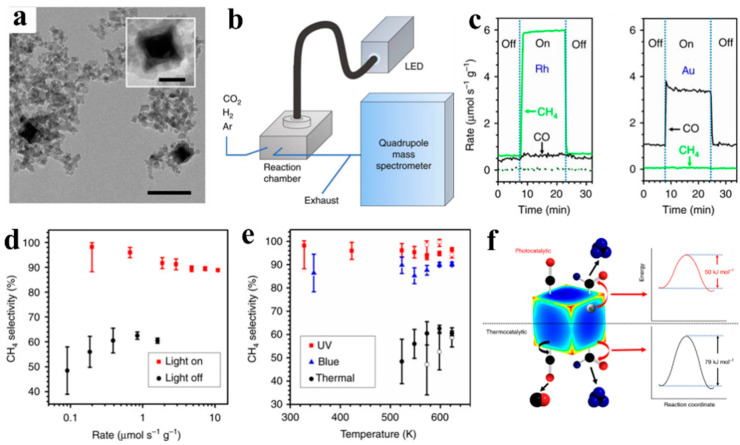
(**a**) Transmission electron microscopy (TEM) images of the Rh/Al_2_O_3_ photocatalyst (Scale bar, 100 nm (inset: 25 nm)). (**b**) Schematic of the photocatalytic reaction system. (**c**) Rates of CH_4_(green) and CO (black) production at 350 °C on Rh/Al_2_O_3_ and Al_2_O_3_ under conditions of dark and ultraviolet illumination at 3 W cm^−2^. (**d**) Selectivity toward CH_4_ as a function of overall reaction rates in the dark and under ultraviolet light. (**e**) Selectivity toward CH_4_ of the thermocatalytic and photocatalytic reactions. (**f**) Reaction mechanism on a rhodium nanocube (The black, red, and blue spheres represent carbon atom, oxygen atom, and hydrogen atom.) [83].

Recently, the plasmonic properties of Rh and Au in photocatalytic CO_2_ reduction were investigated by Liu et al. [83]. A Rh/Al_2_O_3_ catalyst was prepared via an impregnation method. Rh nanocube colloids were dropped onto Al_2_O_3_ nanoparticles, and the formed solid was ground and calcined in air (Figure 7a) and then tested in a fixed-bed reactor (Figure 7b). It was found that the CH_4_ and CO production rates for Rh/Al_2_O_3_ were similar at 350 °C without light irradiation. When ultraviolet light (3 W cm^−2^) was introduced, a seven-fold increase in the CH_4_ production rate was detected, while the CO production rate changed only very slightly (Figure 7c). In contrast, plasmonic Au nanoparticles could catalyze only CO production, regardless of the presence of irradiation (Figure 7d). It is also worth noting that the CH_4_ selectivity of unheated Rh nanoparticles was over ~86% or ~98% compared to the dark condition or thermo condition, respectively. And, the reaction rate was double that of the thermocatalytic reaction rate at 350 °C (Figure 7d,e). These experimental results together indicated the selective production of CH_4_ via a photothermal catalytic process. Further kinetic studies, together with DFT calculations, revealed that photo-excited hot electrons generated through the LSPR effect of Rh nanocubes would selectively be transferred into the antibonding orbital of the CHO intermediate, which can lower the activation energy of CH_4_ production by ~35% relative to the thermal activation energies (Figure 7f). This study well demonstrates the idea of tuning product selectivity in CO_2_ reduction by using light to excite plasmonic electrons and activate key reaction intermediates.

**Figure 8:**
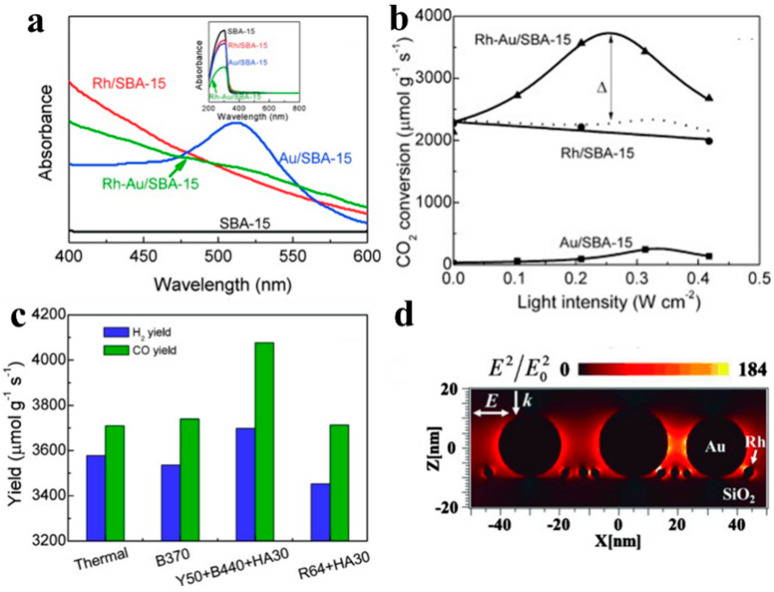
(**a**) UV-Vis spectra of the Rh/SBA-15, Au/SBA-15, and Rh-Au/SBA-15 catalysts. (**b**) Effects of visible light intensity on the performance of catalysts in the dry reforming reaction. (**c**) H_2_ and CO yields without and with light irradiation of different wavelength ranges. (**d**) Cross-sectional views of the electromagnetic field distribution and enhancement simulated for Rh-Au/SBA-15 through a finite-difference time-domain (FDTD) method [11].

In order to achieve an efficient dry reforming reaction (CO_2_ + CH_4_ → 2CO + 2H_2_), Ye et al., used plasmonic nanoparticles to promote the activity of their noble metal-based catalyst (Figure 8) [9]. In this case, a Rh-Au/SBA-15 bimetallic catalyst was synthesized via a subsequent impregnation pathway, with the prepared catalyst showing a strong LSPR corresponding to Au nanoparticles (Figure 8a) [11]. In a flow reactor system with visible light illumination of 420 to 800 nm and a temperature of 500 °C, the activity catalyst was enhanced 1.7 times compared to that observed in the dark. Additionally, the catalytic activity of Rh-Au/SBA-15 was much higher than that of either Rh/SBA-15 or Au/SBA-15 (Figure 8b). UV-Vis spectra and electromagnetic field simulation results confirmed the corresponding relationship between the enhanced dry reforming activity and the LSPR band of Au (Figure 8c,d). This demonstrated the photothermal activity of the dry reforming reaction over the Rh-Au/SBA-15 catalyst and the role of energetic “hot” electrons excited by LSPR in Au, contributing both thermal energy and polarization/activation of CO_2_ and CH_4_ to the catalytic reaction.

**Figure 9:**
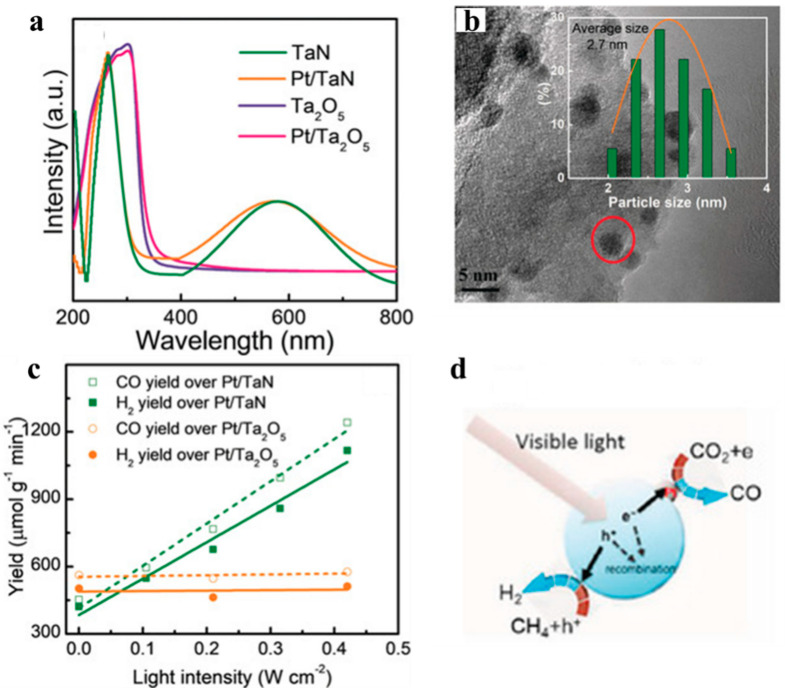
(**a**) UV-Vis spectra of TaN, Ta_2_O_5_, and the Pt-based catalysts. (**b**) TEM images of Pt/TaN. (**c**) Catalytic performance of Pt/TaN and Pt/Ta_2_O_5_ in the dry reforming reaction. (**d**) Mechanistic illustration of the visible-light-assisted dry-reforming reaction over Pt/TaN catalysts [84].

In another work from the same group, TaN was used as a support for loading Pt nanoparticles, chosen due to its intense light absorption over a range of wavelengths (Figure 9a) [84]. This Pt/TaN catalyst was prepared via a simple impregnation method, resulting in an average Pt nanoparticle diameter of approximately 2.7 nm (Figure 9b). The Pt/TaN catalyst was also tested in the dry reforming reaction under visible light irradiation at 500 °C, which revealed that the CO_2_ conversion rate of the catalyst was 2.7 times that observed in the dark. In a control experiment employing Pt/Ta_2_O_5_ as a catalyst, the catalytic activity remained nearly constant even under visible light illumination (Figure 9c). Using DFT calculation results, the polarity of TaN was revealed to be a critical parameter for the efficient separation of electron–hole pairs generated in the photothermal excitation. In conclusion, this study emphasized the importance of a support material’s optical absorption properties in photothermal reactions (Figure 9d).

**Figure 10:**
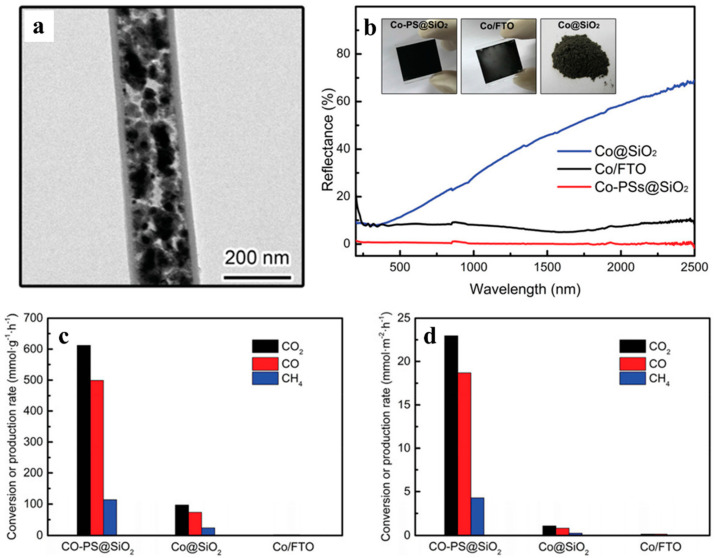
(**a**) Cross-sectional TEM images of Co-PS@SiO_2_. (**b**) Diffuse reflectance spectra of each cobalt catalyst material. Insets show the corresponding photographs of these samples. (**c**,**d**) Conversion rates of reagents and production rates of products from the catalyst normalized by (**c**) the mass of Co and (**d**) the surface area of Co [70].

The aforementioned plasmonic properties of Pd, Rh, and Au demonstrate the promotion of photocatalytic activity by extending the light absorption range, suppressing the charge-carrier recombination, and forming highly active surface reaction sites. However, the relatively high cost and low abundance of noble metals place a hard limit on their practical application on a global scale. With this in mind, Ozin et al., have recently developed plasmonic cobalt superstructures capable of achieving nearly 100% sunlight absorption while acting as photocatalysts for efficient CO_2_ reduction. [70] This cobalt plasmonic superstructure was composed of a nanoporous, needle-like structure containing Co metal cores and an enclosing silica shell, Co-PS@SiO_2_ (Figure 10a). For comparison, Co@SiO_2_ and Co/FTO samples were also prepared via traditional impregnation methods. The diffuse reflectance spectra of different catalysts were characterized as shown in Figure 9b. It was demonstrated that the silica shell can prevent the nanoneedle structure from collapsing and confine the growth of Co nanocrystal. As a result, more active sites will be preserved, and the photon path length can be simultaneously increased through multiple reflection. Through detailed experimental comparisons, this study concluded that the strong light absorption of Co metal combined with the antireflection of the nanoarray structure contributes to the broadband optical absorption across the entire solar spectrum. (Figure 10b). The photocatalytic activity was evaluated in a batch reactor system operating at atmospheric pressure with a 1:1 feed ratio of H_2_/CO_2_. The catalytic testing results of the three aforementioned catalysts are presented in Figure 10c,d. The CO_2_ conversion rate of Co-PS@SiO_2_ was about 0.6 mol g_Co_^−1^ h^−1^ (normalized to the mass of Co), which is 6 times that of Co@SiO_2_ and 277 times that of Co/FTO (Figure 9a). When normalized by surface area, these respective activity increases were 20 and 151 times greater (Figure 10b). Moreover, the CO selectivity of Co-PS@SiO_2_ was higher than that of Co@SiO_2_, which is also consistent with the higher local catalyst temperature caused by the better light absorption ability. The ability of Co-PS@SiO_2_ to fully harvest solar energy demonstrates the applicability of base metal plasmonic superstructures in other solar energy harvesting systems.

## 3. Summary and Outlook

In this review, we have focused on recent developments in solar-energy-driven CO_2_ hydrogenation reactions, from the design of catalysts to the structure of active sites and mechanistic investigations. To date, several strategies have been developed to improve the catalytic efficiency and overcome the challenges facing CO_2_ reduction. For the purpose of utilizing light energy, localized surface plasmon resonances of metal particles (Pd, Rh, Ni, Co, etc.) and vacancies have been proven as two simple and efficient approaches to widening the light absorption range of light-driven catalyst materials. The presence of metal particles not only enhances the optical absorption capability but also provides active sites for the activation of H_2_ and CO_2_. As a surface catalytic reaction, the presence of anion vacancies can both improve the adsorption of CO_2_ molecules and lower the activation energy associated with their reduction. Specialized nanostructures have also been constructed to increase the utilization of light, including photonic crystals that can enhance light trapping. Indium-based oxides are found to have excellent performance in CO_2_ hydrogenation because of their flexibility in terms of morphologies, phases, and surface-active sites. Though good progress has been made, several challenges must still be overcome before practical applications of these catalysts becomes possible.

First, the catalytic activity of many reported catalysts is still limited; therefore, improving nanoscale architectural design, bandgap engineering, and broad spectral adsorption appears to be particularly important. Many experimental and theoretical studies have been undergoing to study the relationship between spectral adsorption properties and catalytic performance; however, the lack of enough in-situation or operando activities make it a challenge to discover the internal mechanism.

Second, the cost of many of these catalysts is prohibitively high. Most of the developed catalysts contain precious metals; however, increasing atomic efficiency through alloying or the development of single atom catalysts may be offering some improvement. Moreover, it is also important to design some non-noble metals and noble metals (bimetal)/semiconductor catalysts in the process of photocatalytic CO_2_ reduction, such as Cu, Bi, et al.

Finally, the industrial-scale preparation of these catalysts and the developments of specialized equipment are still relatively scarcely reported. In a word, with the continuous development of broadband, highly absorbing materials, and increased understanding of photochemical and thermal properties in nanostructured catalysts, the future of photothermal catalysis offers many interesting opportunities for CO_2_ capture and conversion technologies. The combining of photocatalytic and thermocatalytic approaches will doubtlessly provide new development opportunities for efficient CO_2_ hydrogenation.

## Data Availability

Data will be made available on request.
